# Medication Management Apps for Diabetes: Systematic Assessment of the Transparency and Reliability of Health Information Dissemination

**DOI:** 10.2196/15364

**Published:** 2020-02-19

**Authors:** Zhilian Huang, Elaine Lum, Josip Car

**Affiliations:** 1 Centre for Population Health Sciences Lee Kong Chian School of Medicine Nanyang Technological University Singapore Singapore; 2 NTU Institute for Health Technologies Interdisciplinary Graduate School Nanyang Technological University Singapore Singapore; 3 Institute of Health and Biomedical Innovation Queensland University of Technology Brisbane Australia; 4 School of Clinical Sciences Faculty of Health Queensland University of Technology Brisbane Australia

**Keywords:** health apps, digital health, diabetes, privacy, evidence-based guidance

## Abstract

**Background:**

Smartphone apps are increasingly used for diabetes self-management because of their ubiquity and ability to help users to personalize health care management. The number of diabetes apps has proliferated in recent years, but only a small subset of apps that pose a higher risk are regulated by governmental agencies. The transparency and reliability of information sources are unclear for apps that provide health care advice and are not regulated by governmental agencies.

**Objective:**

This study aimed to assess the transparency and reliability of information disseminated via diabetes apps against 8 criteria adapted from the Health On the Net code of conduct (HONcode) principles.

**Methods:**

English-language diabetes-related terms were searched on a market explorer (42matters) on June 12, 2018. Apps with medication and blood glucose management features were downloaded and evaluated against the App-HONcode criteria adapted from the 8 HONcode principles: authoritative, complementarity, privacy, attribution, justifiability, transparency, financial disclosure, and advertising policy. Apps were profiled by operating platforms (ie, Android and iOS) and the number of downloads (ie, Android only: ≥100,000 downloads and <100,000 downloads).

**Results:**

A total of 143 apps (81 Android and 62 iOS) were downloaded and assessed against the adapted App-HONcode criteria. Most of the apps on the Android and iOS platforms fulfilled between 2 and 6 criteria, but few (20/143, 14.0%) apps mentioned the qualifications of individuals who contributed to app development. Less than half (59/143, 39.2%) of the apps disclaimed that the information provided or app functions do not replace the advice of the health care provider. A higher proportion of iOS apps fulfilled 5 or more App-HONcode criteria compared with Android apps. However, Android apps were more likely to have the developer’s email listed on the app store (Android: 75/81, 98%; and iOS: 52/62, 84%; *P*=.005) compared with iOS apps. Of the Android apps assessed, a significantly higher proportion of highly downloaded apps had a privacy and confidentiality clause (high downloads: 15/17, 88%; and low downloads: 33/64, 52%; *P*=.006) and were more likely to discuss their financial sources (high downloads: 14/17, 82%; and low downloads: 32/64, 50%; *P*=.03) compared with apps with a low number of downloads.

**Conclusions:**

Gaps in the disclosure of the developer’s qualification, funding source, and the complementary role of the app in disease management were identified. App stores, developers, and medical providers should collaborate to close these gaps and provide more transparency and reliability to app users. Future work can further examine the consent-seeking process for data collection, data management policies, the appropriateness of advertising content, and clarity of privacy clause of these apps.

## Introduction

### Background

Smartphone apps, software designed to run on mobile devices, have integrated into many aspects of daily lives and are increasingly used for disease management. For example, there are more than 2000 consumer apps to choose from to self-manage diabetes [1]. In contrast to Web-based information accessed from the computer, apps can help the user to conveniently access information with their portability and improved technological capabilities, such as increased human interaction, interoperability with other devices, and easy data collection. Health apps can have a multitude of uses, including offering advice on healthy living, communication with health care providers, and providing decision support through granular biometric data collection (eg, blood glucose) [2-4]. The adoption of health apps for chronic conditions such as diabetes is expected to grow [5]. There are currently 2.5 billion smartphone users in the world [6] and more than 300,000 health apps available for consumer download [5].

One main reason for the proliferation of health apps is the low barrier of entry for app developers to publish apps [3]. Although the 2 major app stores (ie, Apple and Google Play) review apps before publication (Multimedia Appendix 1), many apps that do not conform to the prereview checklist fall through the cracks and are published. The regulation of app stores also falls outside the purview of governmental agencies, such as purview of the Food and Drug Administration (FDA) [7]. Only a small subset of apps that can pose a higher risk and meet the regulatory definition of *device* are regulated by the FDA [8-10]. Therefore, apps with inaccurate content or advertisements may still be published and be available to consumers [11-13]. The lack of transparency regarding an app’s source of content may compromise the reliability of the information it disseminates [9,14,15] and can potentially mislead or cause harm to patients who have low health literacy [16].

Concerns over the credibility and reliability of Web-based health information sources began to surface in the early days of internet usage [17,18]. The Health On the Net code of conduct (HONcode), which covers 8 principles (ie, authoritative, complementarity, privacy, attribution, justifiability, transparency, financial disclosure, and advertising policy) for website certification, was developed to help guide and standardize the reliability of health and medical information published on the internet [19]. Websites that were certified by the HONcode were assessed to be of higher quality and may reduce consumers’ burden of searching for good-quality websites [20,21].

### Objectives

As part of a larger study investigating the medication management features of diabetes apps [22], this study aimed to assess the transparency and reliability of information disseminated via these apps against 8 criteria adapted from the HONcode principles.

## Methods

### Development of App Assessment Criteria

We adapted the 8 HONcode principles (ie, authoritative, complementarity, privacy, attribution, justifiability, transparency, financial disclosure, and advertising policy) and termed it the App-HONcode criteria to suit the context of health apps and apps assessment. The initial versions of our criteria were piloted with highly downloaded (≥100,000 downloads) diabetes apps to refine and improve the clarity of the assessment criteria. Unclear statements were discussed among the app assessment team members until a consensus was reached. The *not applicable (N/A)* option was included for the assessment of *attribution* and *justifiability* to account for apps that did not provide health information within the app. The final assessment criteria are shown in (Table 1).

**Table 1:** Adapted Health On the Net code of conduct criteria for health app assessment.

Attribute	HONcode^a^	App-HONcode criteria	Options
Authoritative	The qualifications of the authors are indicated.	Does the app indicate the qualifications of specific individuals who developed or contributed to the development of the app?	Yes or no
Complementarity	Information should support, not replace, the doctor-patient relationship.	Is there a disclaimer stating or which implies that the information provided and/or app functions do not replace the health care provider’s advice?	Yes or no
Privacy	Respect the privacy and confidentiality of personal data submitted to the site by the visitor.	Is there a privacy and confidentiality clause in the app?	Yes or no
Attribution	Cite the source(s) and date of published information on medical and health pages.	Are information sources in the app cited?	Yes, no, or N/A^b^
Justifiability	Justifiability: site must back up claims relating to benefits and performance.	Are the claims relating to benefits and performance in the app description backed up by evidence? (Answer *N/A* if there are no claims)	Yes, no, or N/A
Transparency	Accessible presentation and accurate email contact.	Are the developers contactable by email?	Yes or no
Financial disclosure	Identify funding sources.	Does the app indicate any funding sources? (*Yes* if the app is managed by a registered commercial company)	Yes or no
Advertising policy	Clearly distinguish advertising from editorial content.	Are advertorials distinguishable from content of the app?	Yes, no or no advertising

^a^HONcode: Health On the Net code of conduct.

^b^Not applicable.

### App Selection and Assessment

As the app assessments were part of a larger study investigating medicines management functionalities of diabetes apps, a more detailed description of app selection and assessment can be found in another paper [22].

#### App Search Strategy

Diabetes terms in the English language were searched in the Google Play and Apple app stores via an app market explorer, 42matters [23], on June 12, 2018, to identify apps that were marketed for diabetes self-management. The search terms *(Diabetes OR Diabetic OR Diabetics) OR (glucose OR glycaemic OR glycemic OR blood sugar OR HbA_1c_ OR A_1c_) OR insulin* were used to search app descriptions, and a list of app titles with descriptions was produced for screening.

#### App Selection

A random sample of 100 apps was first screened by 2 researchers (ZH and MLT) to ensure consistency in interpretation of the inclusion and exclusion criteria listed below. Differences in interpretations were resolved via consensus discussion with 2 other team members (EL and GJ). Random samples of apps were rescreened until an interrater agreement of above 80.0% was achieved.

#### Inclusion Criteria

The inclusion criteria were as follows: Apps with medication self-management features (ie, medication scheduling, reminders, tracking, information provision, and adherence review), apps with any blood glucose logging features, apps in the English language, free apps, and apps requiring payment.

#### Exclusion criteria

The exclusion criteria were as follows: Patient health portals linking to patients’ electronic health records, apps that were not updated after January 1, 2017, intended only for health care professionals, insulin calculators/bolus correctors only, apps with exclusive blood glucose monitoring device tie-in requirement (ie, not permitting manual entry of blood glucose values), apps duplicated on the same platform, apps with regional restrictions, and technical problems (eg, crashes, screen hangs, unable to login, and unable to download).

#### App Assessments

Included apps were evaluated against the adapted App-HONcode criteria. In total, 5 team members (ZH, EL, GJ, CT, and MLT) underwent a calibration exercise to ensure consistency in criteria interpretation before the app assessments. Apps available on the iOS and Android platforms were treated as unique apps and assessed on both platforms because of possible differences in versions across platforms. The number of *yes* responses was summed up for each app to determine the number of App-HONcode criteria met. *N/A* responses for *attribution* and *justifiability* were treated as *not meeting the criteria* for a more conservative approach, whereas the *no advertising* response for *advertising policy* was treated as a *yes* (conforming to this principle).

### Statistical Analyses

Apps were grouped by operating platform (ie, Android and iOS) and profiled according to each App-HONcode criterion using descriptive statistics. Only Android apps were further classified by the number of downloads (ie, ≥100,000 downloads and <100,000 downloads), as information on the number of downloads was not available for iOS apps. Pearson chi-square test was used for comparisons between groups, and a 2-tailed Fisher exact test was used where the expected count was less than 5 in a group. Statistical significance was set at a *P* value of less than .05. All analyses were performed using SPSS (version 22; IBM Corp).

## Results

### App Screening

We identified and downloaded 351 apps (191 Android and 160 iOS) after app title and description screening, of which 143 apps (81 Android and 62 iOS) met the inclusion criteria and were assessed against the app assessment criteria. The detailed app search results can be found in our study [22].

### Characteristics of Included Apps

**Figure 1:**
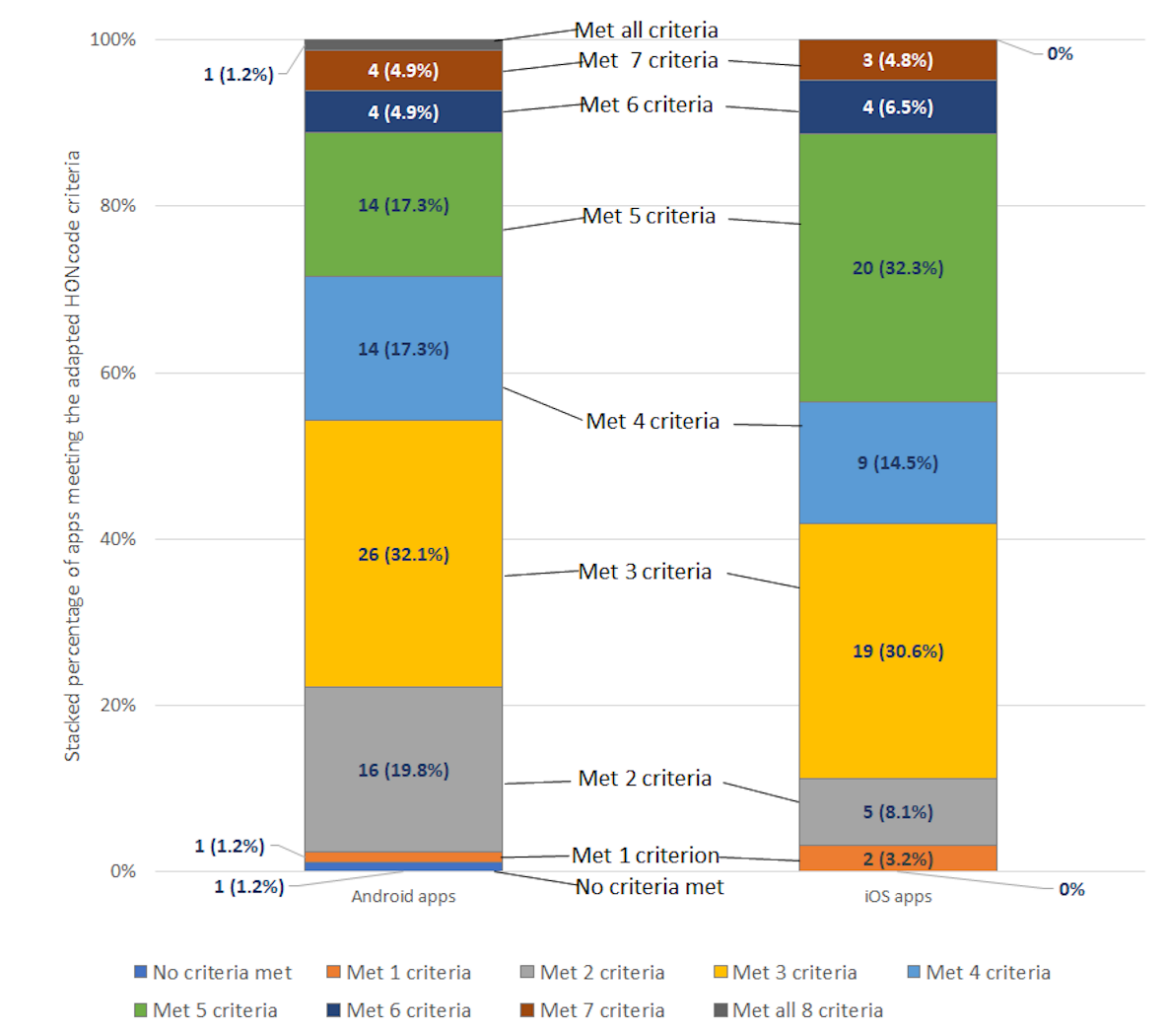
The number and proportion of diabetes apps meeting the App-HONcode app criteria. The number of Yes responses were summed up for each app to determine the number of app-HONcode criteria met. N/A responses for attribution and justifiability were treated as no, whereas the no advertising response for advertising policy was treated as a yes. HONcode: Health On the Net code of conduct.

The number and proportion of the assessed apps meeting the App-HONcode criteria are shown in Figure 1. Most of the apps on the Android and iOS platforms fulfilled between 2 and 5 criteria; 1 Android app met all 8 criteria, whereas another did not meet any criteria. A higher proportion of apps published on the iOS platform met more App-HONcode criteria compared with apps on the Android platform. For example, 43.6% of iOS apps met at least five App-HONcode criteria compared with 28.3% of Android apps.

The profile of app attributes grouped by platform is shown in Table 2. Few (20/143, 14.0%) apps mentioned the qualifications of individuals who contributed to app development. Less than half (56/143, 39.2%) of the apps had a disclaimer stating that the information provided/app functions do not replace a health care provider’s advice, and approximately two-thirds (93/143, 65.0%) of apps had a privacy and confidentiality clause. Of the apps providing health or medical information or made claims on its efficacy, only one-third cited their information sources (15/42, 36%) and/or backed up the claims relating to benefits and performance in the app by evidence (7/23, 30%).

**Table 2:** Profile of app attribute grouped by platform.

HONcode^a^ principle	App-HONcode criteria	All apps, n (%)	Android, n (%)	iOS, n (%)	*P* value
Authoritative	Does the app indicate the qualifications of specific individuals who developed or contributed to the development of the app?	20 (14.0)^b^	9 (11)^c^	11 (18)^d^	.33
Complementarity	Is there a disclaimer stating or which implies that the information provided and/or app functions do not replace the health care provider’s advice?	56 (39.2)^b^	27 (33)^c^	29 (47)^d^	.12
Privacy	Is there a privacy and confidentiality clause in the app?	93 (65.0)^b^	48 (59)^c^	45 (73)^d^	.11
Attribution	Are information sources in the app cited?	15 (34)^e,f^	8 (36)^e,g^	7 (32)^e,h^	>.99^i^
Justifiability	Are the claims relating to benefits and performance in the app description backed up by evidence? (Answer *not applicable* if there are no claims)	7 (30)^e,j^	4 (29)^e,k^	3 (33)^e,l^	>.99^i^
Transparency	Are the developers contactable by email?	131 (91.6)^b^	79 (98)^c^	52 (84)^d^	.005^m^
Financial disclosure	Does the app indicate any funding sources? (*Yes* if the app is managed by a registered commercial company)	88 (61.5)^b^	46 (57)^c^	42 (68)^d^	.23
Advertising policy	There are no advertisements in the app	119 (83.2)^b^	64 (79)^c^	55 (89)^d^	.18
	Are advertorials distinguishable from content of the app?	18 (75)^n,o^	12 (71)^n,p^	6 (86)^n,q^	.63

^a^HONcode: Health On the Net code of conduct.

^b^N=143.

^c^N=81.

^d^N=62.

^e^Not applicable removed before statistical analysis and percentage computation.

^f^N=44.

^g^N=22.

^h^N=22.

^i^Two-tailed *P* value calculated using Fisher exact test, as the expected count is less than 5 in at least one group.

^j^N=23.

^k^N=14.

^l^N=9.

^m^Statistical significance *P*<.05 in the comparison between Android and iOS app features.

^n^Percentage is computed by dividing the number of apps with distinguishable advertisements with the total number of apps with advertisements.

^o^N=24.

^p^N=17.

^q^N=7.

There were no significant differences between the Android and iOS platforms in the proportion of apps fulfilling each criterion except for the principle of *transparency*. Android apps had a significantly higher proportion of apps with the developer’s email listed on the Google play store compared with apps listed on the Apple store (Android: 75/81, 98%; and iOS: 52/62, 84%; *P*=.005). More than half (88/143, 61.5%) of the apps disclosed funding sources. Finally, most 119/143, 83.2%) of the assessed apps did not have advertisements; of apps with advertisements, three-fourths (18/24, 75%) were distinguishable from the content of the app.

### Android Apps by Downloads

Table 3 shows the profile of app attribute grouped by a low (<100,000 downloads) and high number of downloads (≥100,000 downloads) for Android apps. There were no significant differences between apps with a low and high number of downloads in terms of the *authoritative, complementarity, attribution, justifiability, transparency,* and *advertising policy*. A significantly higher proportion of highly downloaded apps had a privacy and confidentiality clause (high downloads: 15/17, 88%; and low downloads: 33/64, 52%; *P*=.006) and were more likely to discuss their funding sources (high downloads: 15/17, 82%; and low downloads: 32/64, 50%;

**Table 3:** Profile of Android app attributes grouped by the number of downloads.

HONcode^a^ principle	App-HONcode criteria	All Android apps, n (%)	<100,000 downloads, n (%)	≥100,000 downloads, n (%)	*P* value
Authoritative	Does the app indicate the qualifications of specific individuals who developed or contributed to the development of the app?	9 (11)^b^	8 (13)^c^	1 (6)^d^	.68^e^
Complementarity	Is there a disclaimer stating or which implies that the information provided and/or app functions do not replace the health care provider’s advice?	27 (33)^b^	18 (28)^c^	9 (53)^d^	.08
Privacy	Is there a privacy and confidentiality clause in the app?	48 (59)^b^	33 (52)^c^	15 (88)^d^	.006^f^
Attribution	Are information sources in the app cited?	8 (36)^g,h^	7 (37)^g,i^	1 (33.3)^g,j^	>.99^e^
Justifiability	Are the claims relating to benefits and performance in the app description backed up by evidence? (Answer *not applicable* if there are no claims)	4 (29)^g,k^	3 (30)^g,l^	1 (25)^g,m^	>.99^e^
Transparency	Are the developers contactable by email?	79 (98)^b^	63 (98)^c^	16 (94)^d^	.38
Financial disclosure	Does the app indicate any funding sources? (*Yes* if the app is managed by a registered commercial company)	46 (57)^b^	32 (50)^c^	14 (82)^d^	.026^f^
Advertising policy	There are no advertisements in the app	64 (79)^b^	50 (78)^c^	14 (82)^d^	>.99^e^
	Are advertorials distinguishable from content of the app?	12 (71)^n,o^	11 (79)^n,p^	1 (33)^n,q^	.19^n^

^a^HONcode: Health On the Net code of conduct.

^b^N=81.

^c^N=64.

^d^N=17.

^e^Two-tailed *P* value calculated using Fisher exact test, as the expected count is less than 5 in at least one group.

^f^Statistical significance *P*<.05 in the comparison between Android and iOS app features.

^g^Not applicable removed before statistical analysis and percentage computation.

^h^N=22.

^i^N=19

^j^N=3.

^k^N=14.

^l^N=10.

^m^N=4.

^n^Percentage is computed by dividing the number of apps with distinguishable advertisements with the total number of apps with advertisements.

^o^N=17.

^p^N=14.

^q^N=3.

## Discussion

### Principal Finding

We evaluated 143 apps against 8 App-HONcode criteria to assess the transparency and reliability of information disseminated via diabetes apps. Most apps fulfilled between 2 and 5 assessment criteria, and only 1 Android app fulfilled all 8 criteria. Overall, a higher proportion of iOS apps fulfilled more App-HONcode criteria compared with Android apps, although the differences were not significant.

Many apps were not transparent in indicating the content source of the app. More than half of the assessed apps did not fulfill important criteria, such as indicating the qualifications of individuals involved in the app development and disclaiming that the app does not replace health care provider’s advice. This concurs with a study assessing eczema apps, where only 15% of the app developers indicated their qualifications, and 46% disclaimed that the app does not replace the advice of the health care provider [24]. Although it may be challenging to indicate the qualifications of all individuals involved in the development of a complex app, the qualifications of the main content contributors and a representation of their collaborators should minimally be quoted to account for the content source of the app.

Approximately three-fourths of the assessed apps did not provide any health information. This was not surprising, as disease management apps tend to emphasize management aspects rather than educating the patient [1], which, in our view, presents a missed opportunity for patient education, which can be incorporated into apps. Of the apps that provided health information, only one-third cited the source of information. Few of the assessed apps had claims relating to the benefits and performance of the app. However, only one-third of these apps backed the claims with evidence. Apps or any consumer products with unsubstantiated claims have the potential to mislead and cause harm to the undiscerning consumer [16,25]. Therefore, it is imperative for app stores to check the veracity of claims used in the app description before its publication on the app stores.

Most apps had an email of the developer displayed, but the email address does not guarantee access to the app developer. We contacted the developers of apps that had access restrictions, and only 10% responded to our emails (2 emails sent a week apart). This percentage should be higher for apps that are accessible, but there is a possibility of inoperative email addresses being displayed on the app store. App stores should ensure the inclusion of a valid email address for all health apps for consumer inquiries.

Privacy breaches can erode consumers’ trust in the app. Two-thirds of the apps assessed had a privacy and confidentiality clause. This was an improvement from a study published 6 years ago assessing the availability, scope, and transparency of mobile health (mHealth) app privacy policies on 600 commonly used mHealth iOS and Android apps [15]. One explanation for this improvement could be the changes made to the app store policies to improve the quality of apps over the years [26,27]. However, stricter scrutiny is required on the part of the app stores, given the absence of privacy policies in many of the assessed apps. Although there is an improvement in the presence of privacy policy of the English-language apps we assessed, those that are published on other platforms and in other languages may yield different assessment results.

Approximately 40% of the assessed apps did not have their funding sources indicated. The funding source of an app will affect its development, quality, and the services provided. This represents a gap in which app stores can play a role to improve the quality assurance of health apps. Advertisements were not present in 80% of the assessed apps. The proportion of apps with advertisements in our study may be lower, as we assessed the best version of apps requiring in-app payments for feature upgrades. Previous studies have shown that paid apps were not of higher quality compared with free apps [28,29]. Approximately one-third of the in-app advertisements were judged as being nondistinguishable from the content of the app. We did not scrutinize the appropriateness of the advertising content, which may present an additional gap in the quality and reliability of information disseminated via these apps.

The originators of the HONcode recently published an extension for apps—mHONcode—to cover the certification of health information disseminated on apps [30]. This was only available after we completed our app assessments, but there are minimal conceptual differences between our App-HONcode and the mHONcode. Our criteria were worded to minimize the subjectivity of assessment by different researchers.

### Limitations

There were limitations to the study, despite attempts to minimize bias. First, the scope was limited to diabetes management apps because of being part of a larger study investigating the medication management features of diabetes apps. However, our findings are generalizable to other diabetes apps, as the apps were identified using a systematic search and selection strategy. Second, our assessment criteria may have underrated apps that do not provide health information (eg, medication logging apps). Even so, many apps were not transparent in data privacy and in clearly distinguishing the complementarity of the app (ie, not replacing the health care provider’s advice). Third, the assessment may not reflect the current state of the apps because of constant app updates. However, we believe that our findings remain unchanged, as app updates were mainly for bug fixes and feature upgrades. Fourth, we neither assessed issues surrounding data management nor the content of privacy and confidentiality clauses, which may not accurately disclose the sharing of some personal information [31]. Finally, app assessments were subjective to researcher interpretation, although we attempted to reduce researcher bias by piloting the assessment and using a standardized patient profile when interacting with apps.

### Implications and Future Research

The fulfillment of the 8 App-HONcode criteria are actionable, but not many developers may be aware of the need to indicate background information or to check the content of advertisements, as their main aim is to get the app published. App developers and consumers would benefit from the availability of a standardized checklist to assess the information quality of health apps. It would be challenging for governments to regulate all health apps because of its ubiquity, pace of development, and ambiguity in definition. Therefore, app stores should play a larger role in making the transparency and reliability of information dissemination a basic requirement for app publication. As observed from our app assessments, owing to the higher barriers of entry currently set by the Apple app store (see Multimedia Appendix 1), it had a larger proportion of apps that fulfilled more App-HONcode criteria.

As health apps collect a lot more user data than the internet, the consent-seeking process for data collection and data management policies of these apps should be evaluated in the future. The appropriateness of advertising and clarity of privacy clauses should also be checked, for diabetes management and other chronic disease management apps in other languages and on other platforms, to provide a more complete landscape of the transparency and reliability of information disseminated and collected through these apps.

### Conclusions

Our systematic app assessments of the transparency and reliability of health information disseminated in diabetes apps discovered gaps in the disclosure of the developer’s qualification, funding source, and the complementary role of the app in disease management. App stores should play a larger role in scrutinizing app publication, as higher barriers of app entry will lead to the publication of apps with better disclosure of the app’s content source. As the development of the App-HONcode criteria is preliminary, future work can further examine the consent-seeking process for data collection, data management policies, the appropriateness of advertising content, and the clarity of privacy clauses.

## Appendix

Multimedia Appendix 1 Selected pre-review app publication checklist of Apple and Google Play app stores.

## Abbreviations

FDA: Food and Drug Administration
HONcode: Health On the Net code of conduct
mHealth: mobile health
