# Supplementary material for: Medication Management Apps for Diabetes: Systematic Assessment of the Transparency and Reliability of Health Information Dissemination
Source: JMIR Mhealth Uhealth. 2020 Feb 19;8(2):e15364. doi: 10.2196/15364 (PMC7057820; doi:10.2196/15364)
Supplement: Multimedia Appendix 1 [file mhealth_v8i2e15364_app1.docx]

**Multimedia Appendix 1**

Selected pre-review app publication checklist of Apple and Google Play app stores relevant to the manuscript “Medication management apps for diabetes: A systematic assessment of the transparency and reliability of health information dissemination”

**Apple app store**

The pre-review checklist serves as a guidance for developers who wish to publish apps on the Apple app store and can be found at: <https://developer.apple.com/app-store/review/guidelines/>. Apps do not have to meet all criteria to be published on the app store. The checklist covers the following categories:

**1. Safety**

(Includes: Objectionable Content, User Generated Content, Kids Category, Physical Harm, Developer Information and Data Security).

Checklist items intended to safeguard health and safety include:

- Rejection of prank apps with false information and features
- Requiring disclosure on the validation of methodology to support accuracy claims relating to health measurements
- Requiring the user to check with a doctor in addition to using the app and before making medical decisions
- Rejection of medical apps that are not from an approved entity or did not pass regulatory clearance (Including drug dose calculators)
- Rejection of apps that encourage consumption of tobacco and vape products, illegal drugs, or excessive amounts of alcohol, or that encourages minors to consume any of these substances
- Requiring apps to have valid contact information
- Requiring apps to implement appropriate security measures to ensure proper handling of user information collected and prevent its unauthorized use, disclosure, or access by third parties

**2. Business**

(Includes: In-app purchases, Subscriptions, Hardware-Specific Content, Goods and Services Outside of the App, Apple Pay, Advertising)

Checklist items intended to safeguard consumers’ interest in health apps include:

- Disallowing the creation of an interface for displaying third-party apps, extensions, or plug-ins similar to the App Store or as a general-interest collection
- Disallowing artificial manipulation of a user’s visibility, status, or rank on other services unless permitted by that service’s Terms and Conditions
- Disallowing coercing users to rate the app, review the app, download other apps, or perform other similar actions in order to access functionality, content, or use of the app
- Requiring to display all information used to target advertisements (ads) to users without requiring the user to leave the app. Interstitial ads or ads that interrupt or block the user experience must clearly indicate that they are an ad, must not manipulate or trick users into clicking the ad, and must provide easily accessible and visible close/skip buttons large enough for people to easily dismiss the ad

**3. Design**

(Includes: Copycats, Minimum Functionality, Spam, Extensions, Apple Sites and Services, Alternate App Icons, HTML5 Games, Bots, etc.)

Checklist item intended to safeguard consumers’ interest in health apps include:

- Discouraging the creation of multiple bundle IDs of the same app and piling on to a category that is already saturated

**4. Legal**

(Includes: Privacy, Intellectual Property, Gaming, Gambling, and Lotteries, VPN Apps, Mobile Device Management, Developer Code of Conduct)

Checklist items intended to safeguard consumers’ privacy in health apps include:

- Requiring all apps to link their privacy policy in the App Store and within the app in an easily accessible manner
- Requiring apps to clearly and explicitly identify the type of data to be collected, method of data collection, purpose of data usage
- Disallowing apps to use or disclose to third parties data gathered in the health, fitness, and medical research context for advertising, marketing, or other use-based data mining purposes other than improving health management, for the purpose of health research (with permissions) or a direct benefit to that user (such as a reduced insurance premium). App developers must disclose the specific health data that will be collected from the device
- Requiring apps conducting health-related human subject research to obtain appropriate consent from participants and research to secure and provide proof of approval from an independent ethics review board
- Requiring apps to disclose any third parties the app wishes to share data with and how a user can revoke consent and/or request deletion of their data
- Asking for permission to collect user data even if the data is considered to be anonymous and providing an easily accessible and understandable way to withdraw consent
- Disallowing developers to use information from Contacts, Photos, or other APIs that access user data to build a contact database for personal use or for sale/distribution to third parties

*The app **Performance** checklist categories not listed in this document include: App Completeness, Beta Testing, Accurate Metadata, Hardware Compatibility, and Software Requirements

**Google Play Store**

The pre-review checklist serves as a guidance for developers who wish to publish apps on the Google Play store and can be found at: <https://play.google.com/about/developer-content-policy/#!?modal_active=none>. Apps do not have to meet all criteria to be published on the app store.

The checklist covers the following categories:

**1. Restricted content**

(Includes: Child Endangerment, Inappropriate Content, Financial instruments, Gambling, Illegal activities, User Generated Content, Unapproved Substances)

Checklist items intended to safeguard health and safety include:

- Disallowing apps that promote illegal activities such as the sale and purchase of illegal drugs or prescription drugs without prescription, encouraging the use or sale of drugs, alcohol, or tobacco by minors or providing instructions for growing or manufacturing illegal drugs
- Prohibiting the sales of prohibited pharmaceuticals and supplements. The list of products monitored by google can be found here: [www.legitscript.com](http://www.legitscript.com)
- Prohibiting the publication of apps with false or misleading health claims, including claims implying that a product is as effective as prescription drugs or controlled substances
- Prohibiting the sales of non-government approved products that are marketed to be safe or effective for use in preventing, curing, or treating a particular disease or ailment. Products that have been subject to any government or regulatory action or warning are also prohibited
- Prohibiting the sales of products containing human chorionic gonadotropin (hCG) in relation to weight loss or weight control, or when promoted in conjunction with anabolic steroids

**2. Impersonation and Intellectual property**

(Includes: Impersonation, Intellectual Property)

Checklist item intended to safeguard impersonation and intellectual property include:

- Developers are not allowed to falsely suggest an affiliation with another entity

**3. Privacy, security and deception**

(Includes: User Data, Permissions, Device and Network Abuse, Malicious Behavior, Deceptive Behavior, Misrepresentation)

Checklist items intended to safeguard consumers’ privacy and security include:

- Requiring developer to be transparent in data handling (by posting a privacy policy) by disclosing the collection, use, and sharing of the data, and limiting the use of the data to the purposes disclosed, and the consent provided by the user. In cases where users may not expect that their personal or sensitive user data will be required to provide or improve the features of the app, these disclosures must be within the app as a separate clause
- Limiting the collection and use of sensitive data such as personally identifiable information, financial and payment information, authentication information, phonebook, contacts SMS and call related data, microphone and camera sensor data, and sensitive device or usage data
- By handling all personal or sensitive user data securely, including transmitting it using modern cryptography
- Requiring the developer to request permissions to access data and use the data only for purposes that the user has consented to. Apps must be actively registered as the default SMS, Phone, or Assistant handler before prompting users to accept any of the above permissions and must immediately stop the use of the permission when it is no longer the default handler
- Disallowing developers to sell collected data or use alternative methods (including other permissions, APIs, or third-party sources) to derive data attributed to the above permissions
- Disallowing apps to contain false or misleading information or claims, including in the description, title, icon, and screenshots. For example, Apps that feature medical or health-related functionalities that are misleading or potentially harmful
- Requiring developers to present the consent dialog in a clear and unambiguous way (e.g. Affirmative user action to accept)

**4. Monetization and Ads**

(Includes: Payments, Subscriptions and Cancellations, Ads, Ad Network Certification)

Checklist items intended to safeguard consumers’ interests include:

- Disallowing apps that contain deceptive or disruptive ads
- Disallowing ads that simulate or impersonate the user interface of any app, notification, or warning elements of an operating system
- Prohibiting coercing the user to click an ad or submit personal information for advertising purposes before they can fully use an app
- Ensuring that the ads within the app does not interfere with the operation of the device, and is easily dismissible without penalty
- Ensuring that the ads shown within the app is appropriate for the intended audience, even if the content is compliant with Google Play’s policies

**5. Store listings and promotions**

(Includes: App Promotion, Metadata, User Ratings, Reviews and Installs, Content Ratings)

Checklist items intended to safeguard consumers’ interests include:

- Disallowing apps that directly or indirectly engage in or benefit from promotion practices that are deceptive or harmful to users or the developer ecosystem, such as using deceptive ads on websites, apps, or other properties, including notifications that are similar to system notifications and alerts, promotion or installation tactics that redirect users to Google Play or download apps without informed user action, and unsolicited promotion via SMS services
- Disallowing developers to manipulate the placement of any apps in Google Play by inflating product ratings, review, or install counts by illegitimate means, such as fraudulent or incentivized installs, reviews and ratings

**6. Spam and Minimum Functionality**

(Includes: Spam, Minimum Functionality)

Checklist item intended to safeguard impersonation and intellectual property include:

- Developers are not allowed to publish apps that spam users or Google Play, such as apps that send users unsolicited messages or apps that are repetitive or low-quality

*Other checklist categories not listed in this document include: Other Programs, Designing Apps for Children and Families, Ads and Monetization**,** Policy coverage, Enforcement Process, Managing and Reporting Policy, Violations, Updates, and Other Resources.
